# Evaluation, diagnosis, and treatment of lead poisoning in a patient with occupational lead exposure: a case presentation

**DOI:** 10.1186/1745-6673-2-7

**Published:** 2007-08-24

**Authors:** D'souza Sunil Herman, Menezes Geraldine, Thuppil Venkatesh

**Affiliations:** 1 Department of Biotechnology, MLSC, Kasturba Medical College, Manipal University, Manipal, Karnataka, India; 2 National Referral Center for Lead Poisoning in India (NRCLPI), Department of Biochemistry, St. John's Medical College, Bangalore, Karnataka, India

## Abstract

Amongst toxic heavy metals, lead ranks as one of the most serious environmental poisons all over the world. Exposure to lead in the home and the workplace results in health hazards to many adults and children causing economic damage, which is due to the lack of awareness of the ill effects of lead. We report the case of a 22 year old man working in an unorganized lead acid battery manufacturing unit, complaining about a longer history of general body ache, lethargy, fatigue, shoulder joint pain, shaking of hands and wrist drop. Patient had blue line at gingivodental junction. Central nervous system (CNS) examination showed having grade 0 power of extensors of right wrist & fingers. Reflexes: Supinator- absent, Triceps- weak and other deep tendon reflexes- normal. Investigations carried out during the admission showed hemoglobin levels of 8.3 g/dl and blood lead level of 128.3 μg/dl. The patient was subjected to chelation therapy, which was accompanied by aggressive environmental intervention and was advised not to return to the same environmental exposure situation. After repeated course of chelation therapy he has shown the signs of improvement and is on follow up presently.

## Background

Lead is a ubiquitous and versatile metal which has been used by mankind for many years. It ranks as one of the most serious environmental poisons amongst the toxic heavy metals all over the world. Mankind has used it for many years because of its wide variety of applications. Human exposure to lead is from numerous sources and a myriad of pathways including air, food, dust, soil and water. The common sources of lead exposure are use of certain products containing lead such as lead soldered cans, traditional practices such as folk remedies, cosmetics, artisan ceramics, environmental emissions containing lead and very importantly through occupations such as production, use and recycling of lead, lead smelting, refining, alloying and casting, lead acid battery manufacture and breaking, printing, jewellery making [1-5]. Many workers who are working in the lead based industries are ignorant of the ill effects of lead hence do not take proper precaution while handling it, leading to higher level of exposure. This case report emphasizes the management a of lead poisoning case. The following sections provide an overview of the evaluation, diagnosis and treatment.

## Case presentation

We present a case of twenty-two-year old male admitted to our hospital with the complaints of pain in the upper abdomen, decreased sleep and appetite, general body ache, tiredness, shoulder joint pain, shaking of hands, and wrist drop. On examination he was noted to have basal metabolic index (BMI): 17.2, Pallor: ++, Coarse tremor: ++, BP: 160/100, Pulse: 78/mt, Blue line at gingivodental junction, grade 0 power of extensors of right wrist & fingers; Intrinsic hand muscles- normal, Other limbs- normal power. Reflexes: Supinator- absent, Triceps- weak, Other deep tendon reflexes- normal, Superficial reflexes- normal, Involuntary movement- tremor, Sensory, Cerebellar, Skull & Spine- normal.

Relevant history revealed that he had been working in an unorganized lead based manufacturing unit since 6 years. He claimed to be ignorant of the ill effects of lead and used to work without taking any precautions.

Investigations carried out during the admission in our hospital showed the following results:

Hemoglobin(Hb): 8.3 g/dl(14–16 g/dl); Total count(TC): 6100 C/cu m (4000–10,000 C/cu m); Differential count(DC): Neutrophils 77% (40–78%), Lymphocytes 21% (20–45%), Monocytes 2% (2–10%); Erythrocyte sedimentation rate (ESR): 8 mm/hr (0–9 mm/hr); Mean corpuscular volume (MSV): 82 fl (76–96 fl); Platelet count: 3.1 lac/cu mm(1.5–4.0 lac/cu mm); Peripheral Smear: Normocytic hypochromic with no basophilic stippling; Blood urea: 42 mg/dl (15–45 mg/dl); Serum creatinine: 0.98 mg/dl (0.6–1.2 mg/dl); Random blood sugar: 103 mg/dl (upto 140 mg/dl); Serum Electrolytes: Sodium 136 mEq/L (135–145 mEq/L), Potassium 4.3 mEq/L (3.8–5.5 mEq/L), Chloride 101 mEq/L (95–105 mEq/L); Test for rheumatoid arthritis and anti nuclear antibody (ANA) negative thyroid function test: normal; Zinc protoporphyrin(ZPP): 148 μg/dl(upto 40 μg/dl) ; Blood lead level(BLL): 128.3 μg/dl (acceptable range 10 μg/dl); Other heavy metal screening: below detectable limit.

In the present study, the blue line at the gums prompted measurement of blood lead levels, which was markedly elevated. The patient had low hemoglobin and high ZPP levels indicating the lead induced adverse effects on hematopoitic system. The symptoms like lethargy, fatigue, peripheral neuropathy and weakness of forearm extensor muscles indicate the effects on the nervous system. Studies have shown that lead inhibits the enzymes δ-aminolevulinic acid dehydratase (ALAD) and ferrochelatse of the heme synthetic pathway thus preventing conversion of ALA to porphobilinogen and inhibits incorporation of iron into the protoporphyrin ring respectively. This results in reduced heme synthesis and elevated levels of the precursor δ-aminolevulinic acid (ALA), which is a weak gamma-aminobutyric acid (GABA) agonist that decreases GABA release by presynaptic inhibition [6,7]. Lead is known to compete with metals like calcium, zinc, iron and that are essential to our body. Lead's ability to substitute for calcium is a factor common to many of its toxic actions. Picomolar concentrations of lead, competes with micromolar concentration of calcium for binding sites on cerebellar phosphokinase C, thereby affecting neuronal signaling [8]. Lead intoxication can affect any part of the central nervous system or peripheral nervous system depending on the level and duration of exposure. Lead enters astroglia and neurons via voltage-sensitive calcium channels [9]. Lead also attacks the peripheral nervous system, which controls the muscle and organs outside the brain. In addition lead causes a decrease in muscle strength and eventually at high doses paralysis sets in. This affects the radial nerve in particular, causing wrist drop. The patient reported in the present study had elevated blood pressure, despite any family history. Several lines of evidence point to the association of blood lead levels with increase in the blood pressure [10].

The detailed clinical investigation in the present study helped us to diagnose the patient having lead toxicity. Lead poisoning continues to be an environmental and public health hazard of global proportions around the world. Exposure to excessive levels of lead in the home and the workplace impose immense costs, affecting adults and children suffering from adverse health effects and impaired intellectual development. Studies have found that the highest levels of environmental contamination were associated with uncontrolled recycling operations and that the most highly exposed adults are those who work with lead [11].

In the present study, the patient was unaware of the ill effects of lead and was handling lead without taking any precautions; he worked without the use of personal protective equipments like mask, gloves and safety glasses even though they were provided. He ate and smoked in the working place, which was having poor housekeeping practices and minimum engineering control, lacking local and general exhaust ventilation and washing facilities culminating into alarmingly high blood lead levels of 128.3 μg/dl. According to United States Occupational safety and Health Administration (OSHA) regulation (29 CFR 1910.1025 App B), workers with single BLL of 60 μg/dl or greater or an average of the last three BLLs or all BLLs over the previous six months at or above 50 μg/dl must be removed from his or her regular job to a place of significantly lower exposure.

The patient was advised to stop his lead related occupation and was subjected to repeated course of chelation therapy using the chelator, D-penicillamine (3-mercapto-D-valine), 25–35 mg/kg body weight/day in divided doses for 3 weeks. Chelation therapy is administered in order to increase the rate of excretion of lead in the short term, by 25 to 30 times the normal, which may otherwise take months to years. Chelating agents competitively bind lead, removing it from biologically active molecules, and the complexes formed are excreted from the body. The administration of the chelation to the patient in the present study was accompanied by aggressive environmental intervention, and the patient was not allowed to return to the same environmental exposure situation.

There are many such unorganized battery manufacturing units operating, where the younger generation have exposed to this toxic heavy metal for many years and lead gets deposited in soft tissues and bones of these individuals making these organs endogenous sources of lead for many years even after these individuals are removed from the ongoing exposure. In the present case the patient was subjected to three courses of chelation therapy with 7 days of gap between the each course. The BLL measured immediately after the each course has shown a decline in the levels. The cessation of chelation therapy for 30 days has increased his BLL. Table 1. This patient was subjected to 4^th ^course chelation therapy 32 days after the 3^rd ^course. After repeated course of chelation therapy, he showed some signs of improvement. He responded with an improvement in Hb to 13.2 g/dl. This was accompanied by significant improvement in wrist drop, shaking of hands and tiredness.

**Table 1:** Blood lead (BPb) and ZPP levels before and during the chelation therapy

	Before Chelation	After 1st course of chelation	After 2nd course of chelation	After 3rd course of chelation	30 days after discontinuing chelation	After 4th course of chelation
ZPP (μg/dl)	148	76	75	50	68	58
BPb (μg/dl)	128.3	60.9	51.5	40.1	95.5	60.3

Though the chelation therapy, removes lead from the blood and soft tissues, upon discontinuation of treatment, it is redistributed from the bony compartment to the blood [12]. This clearly suggests that the chronic lead exposure requires repeated courses of treatment. The patients undergoing chelation therapy require intensive monitoring through out the treatment period and blood lead levels should be estimated at the end of each course and the subsequent therapy should be based on this determination.

## Conclusion

Detailed clinical investigation is of prime importance for identifying lead poisoning cases and while treating the lead poisoning cases the chelation therapy must be accompanied by aggressive environmental intervention. The potential health hazards of lead poisoning still exist and are rising due to the lack of education regarding the dangers of working with lead. In a developing country like India where over 80% of used lead is recycled by unorganized sector, who do not comply with any of the government specified regulations. Some of these are as small as a family owned smelting industry. These are neither registered nor visited by any regulating authorities. The lack of a safe workplace and limited awareness among workers in these unorganized industries has resulted in high blood lead levels. Workers in these industries were observed to have poor personal hygiene during and after work; they were observed in the dining area wearing work clothes and observed working without wearing proper respiratory protection, gloves and mask. The employer had failed in providing proper facilities for the workers. There were no proper storage facilities for street clothes and no separate areas were provided for the removal and storage of the lead-contaminated protective work clothing and equipment.

The regulatory body should make it mandatory to evaluate and create awareness in the worker about the ill effects of lead and should insist on regular health check up to prevent adverse health effects. This preventable environmental health hazard can be tackled only through proper awareness and education and by implementing national and international policies.
